# Searching for New Biomarkers to Assess COVID-19 Patients: A Pilot Study

**DOI:** 10.3390/metabo13121194

**Published:** 2023-12-10

**Authors:** Nikolay V. Goncharov, Piotr P. Avdonin, Natalia G. Voitenko, Polina A. Voronina, Polina I. Popova, Artemy V. Novozhilov, Maria S. Blinova, Victoria S. Popkova, Daria A. Belinskaia, Pavel V. Avdonin

**Affiliations:** 1 Sechenov Institute of Evolutionary Physiology and Biochemistry, Russian Academy of Sciences, Saint Petersburg 194223, Russia; ngvoitenko@gmail.com (N.G.V.); p.a.voron@yandex.ru (P.A.V.); artnov20081@rambler.ru (A.V.N.); d_belinskaya@mail.ru (D.A.B.); 2 Koltsov Institute of Developmental Biology, Russian Academy of Sciences, Moscow 119334, Russia; ppavdonin@gmail.com (P.P.A.); maria.s.blinova@gmail.com (M.S.B.); vipopkova2001@gmail.com (V.S.P.); pvavdonin@yandex.ru (P.V.A.); 3 City Polyclinic No.112, Saint Petersburg 195427, Russia; popova.p.i@yandex.ru

**Keywords:** von Willebrand factor, COVID-19, endothelial cells, diagnostics, biomarker, albumin, esterases, indices

## Abstract

During the initial diagnosis of urgent medical conditions, which include acute infectious diseases, it is important to assess the severity of the patient’s clinical state as quickly as possible. Unlike individual biochemical or physiological indicators, derived indices make it possible to better characterize a complex syndrome as a set of symptoms, and therefore quickly take a set of adequate measures. Recently, we reported on novel diagnostic indices containing butyrylcholinesterase (BChE) activity, which is decreased in COVID-19 patients. Also, in these patients, the secretion of von Willebrand factor (vWF) increases, which leads to thrombosis in the microvascular bed. The objective of this study was the determination of the concentration and activity of vWF in patients with COVID-19, and the search for new diagnostic indices. One of the main objectives was to compare the prognostic values of some individual and newly derived indices. Patients with COVID-19 were retrospectively divided into two groups: survivors (n = 77) and deceased (n = 24). According to clinical symptoms and computed tomography (CT) results, the course of disease was predominantly moderate in severity. The first blood sample (first point) was taken upon admission to the hospital, the second sample (second point)—within 4–6 days after admission. Along with the standard spectrum of biochemical indicators, BChE activity (BChEa or BChEb for acetylthiocholin or butyrylthiocholin, respectively), malondialdehyde (MDA), and vWF analysis (its antigen level, AGFW, and its activity, ActWF) were determined and new diagnostic indices were derived. The pooled sensitivity, specificity, and area under the receiver operating curve (AUC), as well as Likelihood ratio (LR) and Odds ratio (OR) were calculated. The level of vWF antigen in the deceased group was 1.5-fold higher than the level in the group of survivors. Indices that include vWF antigen levels are superior to indices using vWF activity. It was found that the index [Urea] × [AGWF] × 1000/(BChEb × [ALB]) had the best discriminatory power to predict COVID-19 mortality (AUC = 0.91 [0.83, 1.00], *p* < 0.0001; OR = 72.0 [7.5, 689], *p* = 0.0002). In addition, [Urea] × 1000/(BChEb × [ALB]) was a good predictor of mortality (AUC = 0.95 [0.89, 1.00], *p* < 0.0001; OR = 31.5 [3.4, 293], *p* = 0.0024). The index [Urea] × [AGWF] × 1000/(BChEb × [ALB]) was the best predictor of mortality associated with COVID-19 infection, followed by [Urea] × 1000/(BChEb × [ALB]). After validation in a subsequent cohort, these two indices could be recommended for diagnostic laboratories.

## 1. Introduction

The search for diagnostic and prognostic markers, the fastest possible stratification of patients upon admission to hospital, and the assessment of the risk and probability of death in the case of many diseases, including COVID-19, is an urgent task of biochemistry, physiology, and experimental medicine. There are distinct temporal trends in selected routine laboratory parameters between survivors and non-survivors with severe COVID-19 admitted to the intensive care unit (ICU), indicating their importance in the prognosis of clinical outcome [1]. Recent investigations revealed new diagnostic biomarkers of the disease, such as mid-regional pro-adrenomedullin [2,3], monocyte distribution width [4,5], electrolyte imbalances [6], pentameric C-reactive protein [7], and pentraxin-3 [8,9]. Also, several recent studies provided insights on adipokines (chemerin, adiponectin, leptin, apelin, visfatin, resistin, galectin-3) as diagnostic and prognostic markers for the severity of COVID-19 [10,11]. Despite the vast volumes of existing evidence, several gaps exist in our understanding of COVID-19 biomarkers, and the pathophysiological basis on which these markers can foretell prognosis in COVID-19 remains poorly understood. Some studies indicate that the search for new single markers of the disease or increasing the sensitivity of methods for determining already known markers is costly and the quality of diagnostics can be significantly improved if several indicators are combined into one derived index [12,13].

On the other hand, no less expensive, high-tech, and labor-intensive, but more effective in terms of the diagnosis and prognosis of the development of diseases (mainly chronic or developing relatively slowly), are the methods of metabolomic analysis and/or the search for an optimal set of indicators (metabolic profile, fingerprint, cluster, etc.) [14,15,16,17,18,19]. At the same time, for practical purposes, it seems promising to search for simple informative indices, for example, based on the ratio of indicators that are in antiphase over a close time interval [20,21,22,23]. For example, among the well-known indices are the De Ritis ratio, the index of atherogenicity, and the body mass index. In the case of infectious or inflammatory diseases, which include COVID-19, this is the ratio of positive and negative acute phase proteins. Most proposed indices include albumin in the denominator: LDH/[ALB], [Lactate]/[ALB], [Fibrinogen]/[ALB], [Urea]/[ALB], and [CRP]/[ALB] [24,25,26,27,28,29,30,31,32,33]. It is important to note that correlation analysis, regardless of age and comorbidity, showed that the [CRP]/[ALB] ratio negatively correlates with oxygen saturation (SO_2_), spirometric parameters (FEV1, forced expiratory volume in the first second; FVC, forced vital capacity), and lung diffusion values (DLCO, diffusion lung capacity for carbon monoxide) [34].

In our recent work [23], we reported the results of a pilot study of patients with COVID-19, according to which a number of diagnostic indices were obtained that characterize the severity of the patient’s condition and the probability of death. Of particular interest is the new index [Urea] × [MDA] × 1000/(BChEb × [ALB]), which was 10-fold higher in the group of deceased patients than in the group of survivors, and 26-fold higher than the value in the group of healthy elderly people.

One of the key components of hemostasis is the von Willebrand factor (vWF); it is synthesized in endothelial cells (EC) and megakaryocytes, and released into the blood in the form of high-molecular multimeric glycoproteins [35,36]. vWF multimers are cleaved into smaller oligomers by the metalloprotease ADAMTS13 [37]. There are a number of microangiopathies (MAPs) associated with an imbalance in the generation and breakdown and/or clearance of vWF. Thus, in hemolytic–uremic syndrome (HUS), an increase in vWF activity in the blood plasma is caused by an increase in its secretion by the endothelium [38]. In another form of MAP, the formation of antibodies in the body that block the activity of ADAMTS13 leads to the development of thrombotic thrombocytopenic purpura (TTP) [39]. From the vesicles of the Golgi apparatus, specific organelles of EC are formed—Weibel–Palade bodies (WPB), containing vWF [40]. In platelets, vWF is located in α-granules [41]. The secretion of vWF by endothelial cells occurs as a result of the constitutive and regulated exocytosis of WPB [42], as well as the participation of autophagosomes [43]. The causes or conditions for the activation of vWF exocytosis are vessel damage, inflammation, receptor activation, hypoxia, and shear stress [44,45,46]. EC damage caused by SARS-CoV-2 exacerbates the endothelial dysfunction that commonly occurs with aging, hypertension, and obesity. As a result, in patients with COVID-19, the secretion of vWF by the endothelium increases, which, together with other factors in severe forms of this disease, leads to thrombosis in the microvascular bed and ultimately to multiple organ failure and the death of the patient [47]. Due to the extremely important role of vWF in the pathogenesis of both COVID-19 and other diseases in which endothelial function is impaired, indicators such as the content of the vWF antigen and the activity of the vWF-degrading metalloprotease ADAMTS-13 are used to objectively assess the patient’s condition. It should be noted that fluctuations in the values of vWF antigen and ADAMTS-13 activity during COVID-19 occur within relatively narrow limits, which reduces the reliability of the forecast. Therefore, to increase the significance of these indicators, the use of the ratio of these two values as a diagnostic index was proposed [48,49].

The purpose of the presented work is a biochemical analysis of the blood plasma of patients with COVID-19, including the determination of the concentration and activity of vWF and the search for new biochemical markers and their derivatives—diagnostic indices that take into account the previously discovered [23] features of esterase status, oxidative stress, and urea content in this disease. Since the primary endpoint was hospital admission with and without death, one of the main objectives was to compare the prognostic values of some individual and new derived indices.

## 2. Materials and Methods

### 2.1. Chemicals

PBS (pH 7.4) was purchased from Biolot (St. Petersburg, Russia). Diagnostic kits were produced by Randox Laboratories (UK). All other reagents are from Sigma-Aldrich (Burlington, MA, USA).

### 2.2. Patients

The study was conducted in accordance with the Declaration of Helsinki, and approved by the Institutional Ethics Committee of Koltsov Institute of Developmental Biology (Approval No. 70, registration date 25 May 2023). Informed consent was obtained from all subjects involved in the study upon admission to the hospital. Patients with a history of COVID-19 (SARS-CoV-2 was confirmed by RT-qPCR) were retrospectively divided into two groups: survivors (n = 77) and deceased (n = 24). General information about the sample is presented in Table 1. There were no significant differences between men (63) and women (38) in age and degree of lung damage (CT results) among survivors and deceased.

According to clinical symptoms and CT results, the course of disease was mostly regarded as moderate, and, in accordance with the Temporary Guidelines “Prevention, diagnosis and treatment of new coronavirus infection (COVID-19)” of the Russian Ministry of Health (versions 16 and 17), the patients received one of the standard treatment regimens, which included drugs of etiotropic therapy (favipiravir, molnupiravir, remdesivir, interferon-alpha), pathogenic therapy (corticosteroids—dexamethasone, methylprednisolone, budesonide; anticoagulant drugs for enteral or parenteral administration—dabigatran etexilate, unfractionated heparin, enoxaparin sodium), and symptomatic therapy (paracetamol; at fever with t > 38.0 °C for more than 3 days, antibacterial and antimycotic therapies were prescribed according to indications).

### 2.3. Sample Preparation and Biochemical Analysis

Blood samples were collected, processed, and stored in accordance with international guidelines [50]. The first blood sample (1st point) was taken upon admission to the hospital within 12 h, the second sample (2nd point)—within 4–6 days, when, based on the results of initial treatment, the issue of transferring the patient to the ICU was discussed. Blood was collected from the subjects on an empty stomach from the cubital vein into BD Vacutainer vacuum tubes. Serum was tested on the day of blood collection. Plasma was stored at −70 °C until the study. Biochemical parameters were determined using a Sapphire 400 analyzer. Methods for determining concentration of malondialdehyde (MDA) and activity of esterases are described in [23].

### 2.4. Von Willebrand Factor Analysis

To quantify vWF in blood plasma using the standard method, the Technozym vWF:Ag ELISA kit (Technoclone GmbH, Vienna, Austria) was used. The activity of von Willebrand factor in the plasma of patients was determined in the agglutination reaction of lyophilized platelets with ristocetin using a set of reagents from the Renam company (Moscow, Russia, No. AG-5).

### 2.5. Statistical Data Processing

Statistical data processing was carried out using the GraphPad Prism 8.4.3 program. For descriptive statistics, medians with ranges from minimum to maximum and interquartile ranges were used. Testing for normality of distribution was carried out in several ways: D’Agostino-Pearson omnibus normality test, Shapiro–Wilk normality test, and Kolmogorov–Smirnov normality test. To compare two groups of unrelated data, the Mann–Whitney test was used, and for paired comparisons, the Wilcoxon matched-pairs signed rank test was used. In all statistical analyses, the significance level was set to α < 0.05.

## 3. Results

In the examined sample of 63 men admitted to the hospital, 44 survived and 19 died (30%), while out of 38 women, 33 survived and 5 died (13%). To identify differences between those who survived and those who died, we used data from a combined cohort of COVID-19 patients. According to the results of a standard biochemical examination (Table 2), statistically significant differences between those who survived and those who died were obtained in the concentration or activity (in the case of enzyme studies) of potassium (9.5% increase), sodium (2.2% increase), chloride (2.2% increase), direct bilirubin (4-fold increase), albumin (18% decrease), total protein (13% decrease), urea (2.4-fold increase), creatine phosphokinase total (CK-NAC, 6-fold increase) and its MB isoenzyme (CK-MB, 2.4-fold increase), troponin (50-fold increase), and procalcitonin (PCT, 3-fold increase). Strange though it may seem, the CRP level showed no significant differences in our study. Other changes were mostly confirmed in the published data from different laboratories. Thus, rhabdomyolysis causing persistent hyperkalemia and acute kidney injury is increasingly recognized as a complication of acute SARS-CoV-2 infection [51,52,53]. Hypernatremia was found to be 97% specific for a poor outcome in COVID-19 patients [6]. Hyperchloremia was significantly associated with increased risks of kidney injury, endotracheal intubation, and death; however, it was not associated with increased ferritin, CRP, or hemoglobin decreases in critically ill COVID-19 patients [54]. Direct bilirubin was found to be one of the best predictors for mortality in cases with COVID-19, so indirect bilirubin may be considered a good protector against complications of the infection [55,56]. CK-NAC and CK-MB increases signify serious cardiovascular complications in COVID-19 patients [57]. A total protein decrease is not a common change, though albumin decrease, together with urea, troponin, and PCT increase, are usual changes in the routine laboratory biomarkers for the detection of severe COVID-19 disease [1,56,58,59].

The study of esterase status, with the additional determination of the level of MDA in the blood plasma, revealed statistically significant differences in the following indicators (Table 3): BChE activity with ATCh and BTCh as substrates (decrease by 27% and 37%, respectively) and MDA concentration (increase by 1.5-fold).

**Figure 1:**
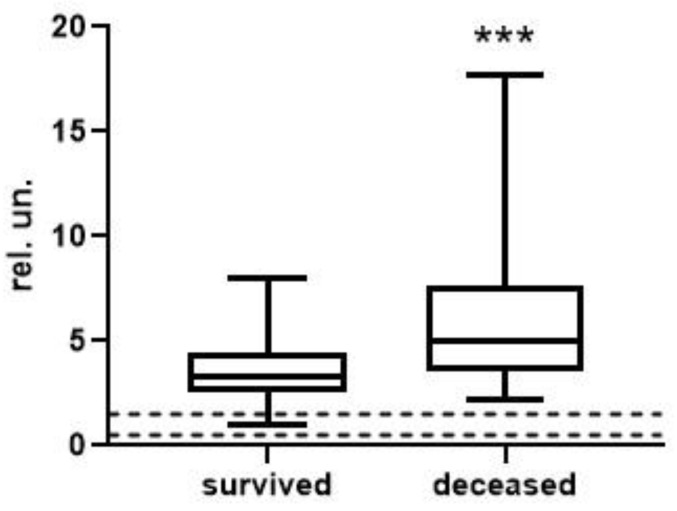
vWF antigen in survivors and deceased at the 1st time point (***, differences are statistically significant, *p* = 0.0002).

**Figure 2:**
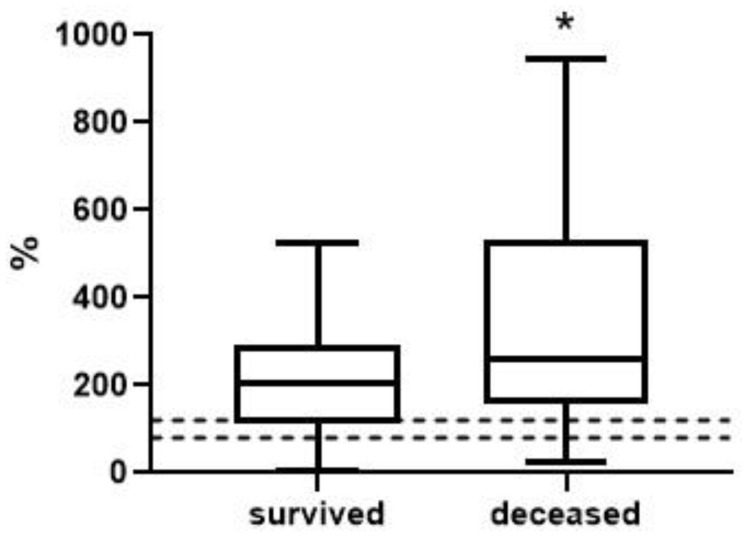
vWF activity in survivors and deceased at the 1st time point (*, differences are statistically significant, *p* = 0.0419).

**Figure 3:**
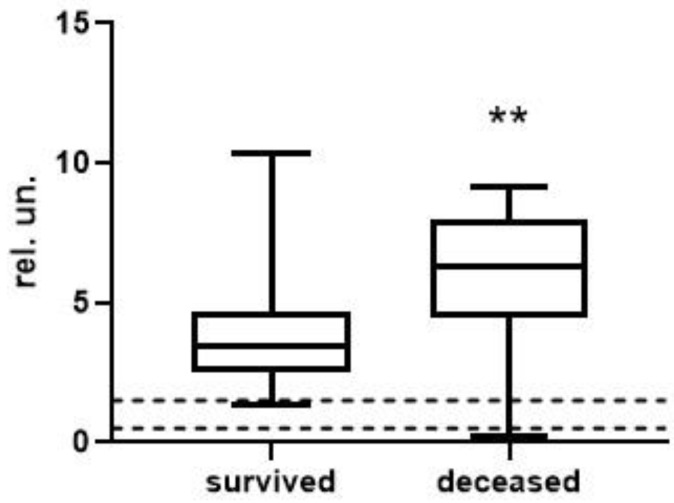
vWF antigen in survivors and deceased at the 2nd time point (**, differences are statistically significant, *p* = 0.0024).

**Figure 4:**
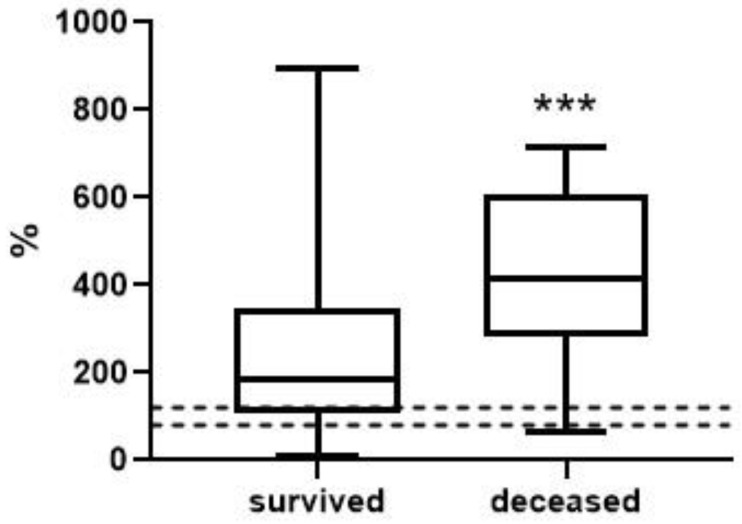
vWF activity in survivors and deceased at the 2nd time point (***, differences are statistically significant, *p* = 0.0009).

To analyze the level and dynamics of changes in vWF antigen and activity, we took the results of measurements at the first time point (upon admission to the hospital), as well as measurements at the second time point—upon discharge from the hospital or upon transfer to the ICU (available for 77 patients, of whom 63 survived and 14 died). A graphical representation of the results of vWF antigen and activity is given in Figure 1, Figure 2, Figure 3 and Figure 4 (the dotted line indicates the normal range of parameters).

At the first time point, the median values of the vWF antigen in the group of deceased were significantly higher by 1.5-fold (*p* < 0.001). vWF activity is also higher in this group, but the changes are less pronounced—only 26% (*p* < 0.05). At the second time point, the concentration of the vWF antigen in the group of deceased was 1.8-fold higher (*p* < 0.01), and changes in activity were much more pronounced compared to the first point—2.2-fold (*p* < 0.001).

**Figure 5:**
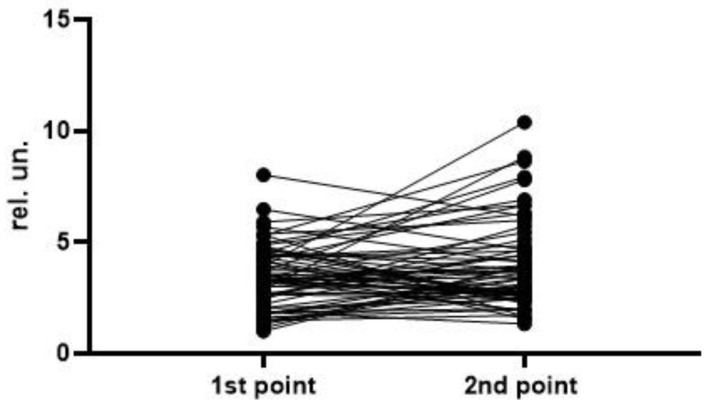
Dynamics of the vWF antigen level in the group of survivors (*p* = 0.0933).

**Figure 6:**
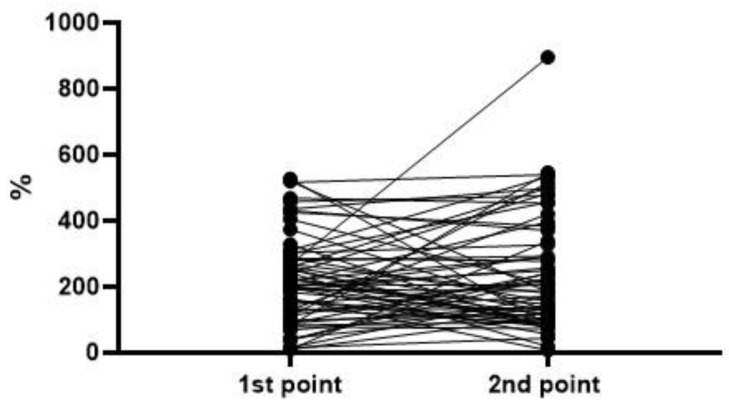
Dynamics of the vWF activity in the group of survivors (*p* = 0.4686).

**Figure 7:**
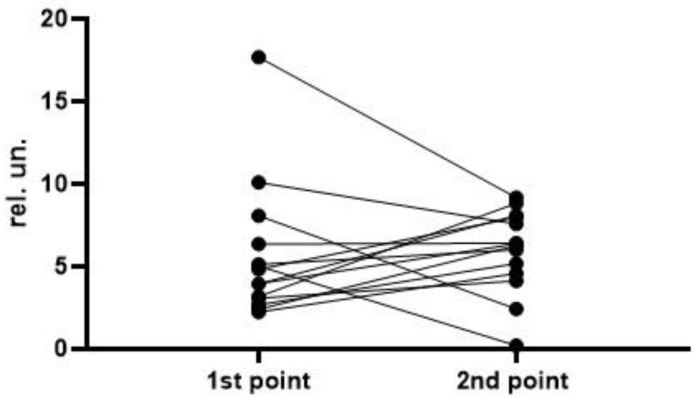
Dynamics of the vWF antigen level in the group of deceased (*p* = 0.6257).

**Figure 8:**
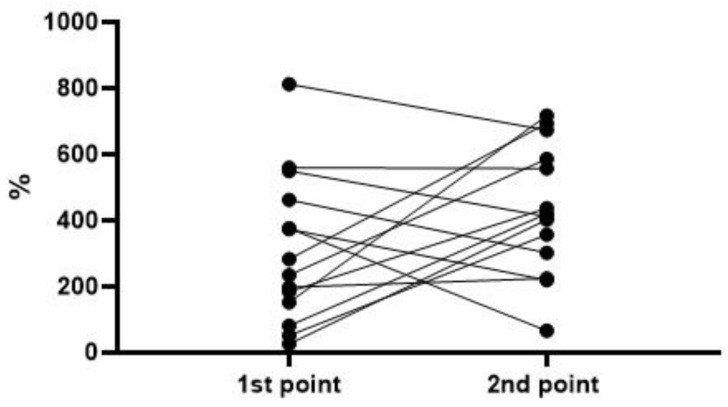
Dynamics of the vWF activity in the group of deceased (*p* = 0.1353).

To assess the dynamics of changes in vWF antigen and activity, 77 and 76 pairs of results were available, respectively. The Wilcoxon test did not reveal significant differences in the parameters in the groups of survivors and deceased. In both groups, one can see both an increase in the antigen and activity, and a decrease; on average (according to medians), both parameters did not show dynamics (Figure 5, Figure 6, Figure 7 and Figure 8). This fact seems extremely important, since it indicates the reliability of the data obtained at the first point (upon admission) for the formation of a prognosis and for the stratification of the patients.

The diagnostic indices using previously identified [23] and new correlations with vWF are presented in Table 4.

Indices with vWF activity are inferior in efficiency to indices with vWF antigen. If the numerator is the product of the concentration of urea and vWF antigen, then, in the deceased group, the index was 7-fold higher (*p* < 0.0001); when using activity, the index differs by 6-fold (*p* < 0.001). If the vWF antigen is in the numerator, then, in the deceased group, the index is on average 3.5-fold higher (*p* < 0.0001); when using activity, the difference remains 3.4-fold, but the statistical significance decreases (*p* < 0.01). The introduction of ferritin concentration into the numerator does not lead to an improvement in diagnostic indices. Therefore, the best indices with vWF should be recognized as [Urea] × [AGWF] × 1000/(BChEb × [ALB]) and [Urea] × ActWF × 1000/(BChEb × [ALB]). In terms of their characteristics, they significantly exceed the basic index [Urea] × 10/[ALB], but are inferior to the previously identified index [Urea] × [MDA] × 1000/(BChEb × [ALB]), according to which the difference between the groups was 8.9-fold. From the calculated indices, two other promising ones can be identified (*p* < 0.0001): [Urea] × [MDA]/[ALB]—the groups differ by 4.9-fold, and [Urea] × 1000/(BChEb × [ALB])—the groups differ by 4-fold.

At the next stage of investigation, we compared the prognostic value of some individual serum biomarkers and complex diagnostic indices (Table 5). The critical value of the indicator was chosen based on the maximum value of the Likelihood ratio (LR) calculated with GraphPad Prism (as a rule, in this case, specificity prevails over sensitivity). After this, the Odds ratio (OR) was calculated using MedCalc’s Odds ratio calculator. Theoretically, one can choose a different level of critical values (for example, with greater sensitivity); then, the LR and OR characteristics will change.

Almost all of the considered indicators have AUC values >0.8 (the exceptions are CRP and ActWF × 1000/(BChEb × [ALB])). Procalcitonin should be excluded from consideration in this array due to the insufficient number of observations (21 in total); the remaining indicators have from 47 to 53 observations. The maximum LR values were noted for four indices, including [Urea] × 10/[ALB], but the highest values were shown for the indices [Urea] × [AGWF] × 1000/(BChEb × [ALB]) and [Urea] × ActWF × 1000/(BChEb × [ALB]). Moreover, the index with the vWF antigen has a larger AUC than the index with vWF activity. In the deceased group, the chances of seeing the index value [Urea] × [AGWF] × 1000/(BChEb × [ALB]) above critical (we chose 0.96) are 72 times higher than in the group of survivors.

## 4. Discussion

In acute COVID-19 patients, effective clinical risk stratification has important implications for treatment and therapeutic resource distribution. Although some studies have indicated an improvement in prognostication when lactate, procalcitonin, IL-6, etc., were added to the SOFA or APACHE II score, the extent to which this would potentially facilitate clinical decision-making remains elusive [12]. Therefore, the most optimal combination of scoring systems and biomarkers requires further study. In any case, single biomarkers are inadequate for the prognostication of individual patients to bacterial or viral infections [12,13].

COVID-related coagulopathy is a major contributor to the overall burden of COVID-related morbidity and mortality. There is now increasing evidence that the combination of increased thromboembolic events and extensive microvascular thrombosis in the pulmonary circulation and elsewhere in the body (systemic venous thromboembolism) may be one of the key drivers of clinical deterioration and death [60]. Multiple factors are involved in the development of a procoagulant milieu in the lungs and the rest of the body, vWF being the principal one.

The main source of circulating vWF is endothelial cells, while platelet-derived vWF is usually bound to receptors on the membrane [61,62]. According to current opinion, the two main pathogenetic factors that cause alveolar edema in COVID-19 are the actual infection of cells with the SARS-CoV-2 virus and the degranulation of mast cells with the release of many compounds that can affect the functions of cell and basement membranes, glycocalyx, and the integrity of tight junctions [63,64]. Of these compounds, histamine, bradykinin, heparin, tryptase, and cytokines are of the greatest interest from the point of view of the pathogenesis of COVID-19. Histamine and thrombin activate the calcium signaling system and show a pronounced activation of vWF exocytosis, in which several WPBs release the vWF contained in them into special secretory vesicles [65,66]. There is also extensive evidence for the stimulation of vWF secretion by reactive oxygen species (ROS), particularly superoxide anion and hydrogen peroxide [67,68,69,70]. The action of some agonists that activate vWF exocytosis is associated with the generation of ROS in endothelial cells [71,72,73,74].

Coronavirus-induced coagulopathy (CIC) is characterized by hypercoagulability in the onset, while consumption coagulopathy and disseminated intravascular coagulation (DIC) are usually recorded in the later stages of the disease [75]. Sepsis in COVID-19 is usually associated with acute respiratory distress syndrome (ARDS), which, in turn, is a manifestation of multiple organ dysfunction syndrome (MODS) and is pathogenetically caused by disseminated microthrombosis. In COVID-19, microthrombosis initially affects the lungs, causing ARDS, but can cause more complex clinical phenotypes, including a TTP-like syndrome, hepatic coagulopathy, MODS, and combined micromacrothrombotic syndrome. During the coronavirus pandemic, ARDS and pulmonary embolism (PE) frequently coexisted. Based on the theory of two pathways of hemostasis, one can come to the conclusion that ARDS caused by the activation of vWF is microthrombosis of endothelial origin, while PE is a consequence of macrothrombosis caused by the activation of vWF and tissue factor. Therefore, ARDS in COVID-19 is a disseminated microthrombosis caused by a virus, while PE is an accompanying macrothrombosis associated with hospital-acquired vascular injury that is not directly related to viral pathogenesis [76].

In the examined cohort of COVID-19 patients, statistically significant differences in the level of albumin, urea, potassium, sodium, chloride, direct bilirubin, total protein, troponin, PCT, CK-NAC, and CK-MB were revealed between those who survived and those who died. CRP levels showed no significant differences in our study, though other changes were mostly confirmed by different laboratories, according to the published data. Statistically significant differences in the activity of BChE (with two substrates, ATCh and BTCh) and the level of MDA were also revealed. Thus, the data from our pilot studies were confirmed in a larger sample of patients [23]. The level of vWF antigen in the deceased group was 1.5-fold higher at the first point and 1.8-fold higher at the second point. The difference in vWF activity was less pronounced at the first point, but more pronounced at the second point. The increase in vWF activity may be due to thedesialylation of vWF molecules—the cleavage of sialic acid residues from glycosyl residues by neuraminidases [77]. However, indices using the vWF antigen are superior to indices using vWF activity. The best index with vWF is [Urea] × [AGWF] × 1000/(BChEb × [ALB]); the value in the deceased group was 7-fold higher than the value in the group of survivors. This index significantly exceeds the basic index [Urea] × 10/[ALB] in its characteristics, but is still inferior in terms of efficiency to the index [Urea] × [MDA] × 1000/(BChEb × [ALB]). However, given the known technical difficulties and time costs associated with the determination of MDA, the vWF antigen index may be an ideal option for modern diagnostic laboratories. This was confirmed by a comparative analysis of the prognostic values of some single and novel derived diagnostic indices, which are constituted by simple measurement indicators and potentially could be determined by any on-line calculator like MedCalc’s free online Diagnostic test statistical calculator.

According to our data, the highest LR and OR values demonstrated indices [Urea] × [AGWF] × 1000/(BChEb × [ALB]) and [Urea] × ActWF × 1000/(BChEb × [ALB]), but the index with the vWF antigen has larger AUC values. Moreover, the previously derived indices with BChE—[Urea] × [MDA] × 1000/(BChEb × [ALB]) and [Urea] × 1000/(BChEb × [ALB])—and even the basic index [Urea] × 10/[ALB]—demonstrated good combinations of AUC, LR, and OR, being alternative candidates for clinical laboratories with low levels of equipment and/or financial security. As was established in our previous study [23], the index with urea and albumin, widely presented and discussed in the literature, in the group of survivors did not differ from the index of healthy volunteers of the same age, but in the group of deceased patients it significantly increased by about 4-fold. The new indices, identified by us in a pilot study of elderly patients with COVID-19, confirmed its high diagnostic efficiency in a wider cohort of patients of different age categories.

When searching for new indices, it is necessary to take into account the level of availability (from a technical point of view) and cost of the proposed indicators—components of a particular index—for numerous laboratories that do not have the ability to conduct expensive tests to determine the acute-phase markers that are elevated during a cytokine storm (ferritin, procalcitonin, troponin, calprotectin, IL-6, etc. [59,78]). Indices that include antigen concentration or vWF activity level are significantly superior to the basic index [Urea] × 10/[ALB] and can serve as good alternatives if there is the technical ability to carry out appropriate measurements in the absence of such capabilities for more labor-intensive and expensive analytic methods. The presence of a wide range of alternative indices in clinical diagnostics will make it possible in the future to more successfully stratify patients with infectious and/or inflammatory diseases/syndromes of various etiologies and pathogenesis. In accordance with the Temporary Guidelines “Prevention, diagnosis and treatment of new coronavirus infection (COVID-19)” of the Russian Ministry of Health (versions 16 and 17), laboratory diagnostics during hospitalization include, along with a clinical blood test, a study of the level of CRP, coagulograms, and the determination of the level of procalcitonin and NT-proBNP/BNP (B-type natriuretic peptide and its N-terminal fragment). Taking into account the diagnostic characteristics of the indices identified in this work, if they are further confirmed and justified, it will be possible to replace the listed expensive methods of analysis with a relatively inexpensive and easy-to-perform determination of the level of vWF antigen, and at least to include the determination of BChE activity in the list of indicators of clinical analysis on a biochemical analyzer. The derived indices could be easily obtained with an on-line calculator.

In many other countries, the requirements for initial diagnosis on admission to hospital are similar, as are the challenges and opportunities for addressing them. Ultimately, we are talking about simplifying the situation and, at the same time, increasing the effectiveness of therapy. As was shown recently, patients who started treatment with non-invasive mechanical ventilation had relatively low poor prognostic factors, and their mortality was lower [79]. Future research should evaluate whether the timely correction of these imbalances improves clinical outcomes.

## 5. Limitations of the Research

Our study has some limitations. In a pilot monocentric study, it is impossible to satisfy all the stringent requirements of evidence-based medicine, the study population for which has to consist of at least 100 adult patients in each group for comparison [12]. Particular difficulties are associated with the expansion of the sample of patients, the essence of which is to justify the receipt of additional blood samples for additional tests. On the other hand, none of the multicenter randomized trials involving several thousand people are possible without preliminary small-cohort comparative analysis. Lastly, the prognostic values of most biomarkers in COVID-19 are derived from retrospective analyses. Prospective studies are required to validate these markers for guiding clinical decision-making and to facilitate their translation into clinical management pathways.

## 6. Conclusions

In patients with a history of coronavirus infection, the secretion of vWF increases, which leads to thrombosis and can be a cause of death. According to the initial examination upon admission to the hospital, the level of vWF antigen in the deceased group was 1.5-fold higher than the level in the group of survivors. Indices that include vWF antigen levels are superior to indices using vWF activity. The index [Urea] × [AGWF] × 1000/(BChEb × [ALB]) demonstrated the highest AUC, LR, and OR values, and, after additional research, can be recommended for diagnostic laboratories. More simple and easily obtained indices with BChE activity and even the basic index [Urea] × 10/[ALB] demonstrated good combinations of AUC, LR, and OR in a wider cohort of patients of different age categories, proving to be alternative candidates for different clinical laboratories.

## Figures and Tables

**Table 1:** General information about the cohort of patients with COVID-19.

	Survivors	Deceased
Total patients (101)	77	24
Age (M ± SD)	49.6 ± 10.9	53.8 ± 7.9
Age Me (min, max)	55 (19, 60)	57.5 (35, 62)
CT results, % lung lesion on admission	36 ± 15	56 ± 19
Men (63)	44	19
Age of men (M ± SD)	48 ± 11.8	53.3 ± 7.7
Age of men Me (min, max)	55 (19, 60)	56 (35, 62)
CT results, % lung lesion on admission	36 ± 15	56 ± 17
Women (38)	33	5
Age of women (M ± SD)	51.7 ± 9.2	55.6 ± 9.3
Age of women Me (min, max)	56 (25, 60)	60 (39, 60)
CT results, % lung lesion on admission	36 ± 16	57 ± 26

CT, computed tomography; M, mean; Me, median; SD, standard deviation.

**Table 2:** Biochemical parameters of patients with COVID-19 upon admission to hospital. S—survivors, D—deceased.

Biochemical Values, Normal Ranges	Outcome	n	Median	Range (Min to Max)	Interquartile Range (1–3 Quartiles)	*p* Value
Potassium, 3.5–5.5 mmol/L	S	68 (88%)	3.88	2.40–5.60	3.50–4.22	0.0087
	D	22 (92%)	4.25 **	3.40–6.30	3.80–4.70	
Sodium, 130–150 mmol/L	S	66 (86%)	137.1	105–145.1	135–139	0.0008
	D	22 (92%)	140.0 ***	131–148	138.7–143.1	
Chloride, 95–110 mmol/L	S	66 (86%)	103.6	79.4–137.0	101–105	0.0449
	D	20 (83%)	105.9 *	91.0–119.0	101.7–110.1	
Calcium, 2.20–2.65 mmol/L	S	5 (6%)	2.29	2.15–2.35	2.20–2.32	
	D	0 (0%)	-	-	-	
Direct bilirubin, 0–3.4 µmol/L	S	7 (9%)	2.10	1.50–3.00	1.70–2.90	0.0105
	D	10 (42%)	8.70 *	1.30–76.80	2.88–26.58	
Total bilirubin, 8.5–20.5 µmol/L	S	60 (78%)	10.00	4.80–23.40	8.13–12.33	0.0769
	D	16 (67%)	12.55	6.4–45.0	8.5–25.23	
Albumin, 35–52 g/L	S	39 (51%)	37.4	29.0–46.5	34.4–41.2	<0.0001
	D	15 (63%)	30.8 ****	19.9–38.9	28.3–34.4	
Total protein, 66–87 g/L	S	65 (84%)	70.6	45.3–88.2	66.6–74.3	0.0057
	D	19 (79%)	61.6 **	44–83	57.6–70.5	
Glucose, 3.5–6.10 mmol/L	S	68 (88%)	5.36	3.40–18.32	4.81–6.64	0.3383
	D	19 (79%)	6.20	1.00–15.30	4.20–9.10	
Iron, 10–32 µmol/L	S	25 (32%)	7.6	2.3–28.6	3.3–13.0	0.5165
	D	4 (17%)	12.8	2.7–58.7	3.4–49.1	
Ferritin, 20–250 µg/L	S	53 (69%)	482	7.8–3306	190.4–656	0.1311
	D	11 (46%)	616	134–1212	330–963	
Creatinine, 72–127 µmol/L	S	70 (91%)	90.3	61.1–203.8	76.9–104.4	0.5848
	D	23 (96%)	93.1	7.9–279.5	71.5–142.8	
Urea, 2.80–7.20 mmol/L	S	73 (95%)	4.90	2.30–21.0	4.15–7.05	<0.0001
	D	23 (96%)	11.90 ****	3.20–50.50	8.10–24.10	
ALT, 0–50 U/L	S	75 (97%)	30.5	10.7–191	20.6–56.9	0.0553
	D	23 (96%)	42.1	13.0–2504	32.8–50.2	
AST, 0–50 U/L	S	74 (96%)	37.3	16.7–168.6	28.1–55.9	0.1301
	D	23 (96%)	43.7	21.3–4045	33.7–80.0	
GGT, 0–55 U/L	S	26 (34%)	75.5	11.4–429.3	36.75–139.6	0.9630
	D	2 (8%)	-	14.5–178.6	-	
ALP, 30–120 U/L	S	54 (70%)	71.5	31.7–346.8	55.3–103.0	0.0478
	D	10 (42%)	89.6	61.2–198.1	80.7–117.8	
Amylase, 28–100 U/L	S	65 (84%)	54.9	14.4–157.8	40.3–77.0	0.1819
	D	21 (88%)	87.9	15.2–494.2	38.1–119.0	
LDH, 0–248 U/L	S	31 (40%)	262	113–595	208–406	0.0758
	D	2 (8%)	-	455.4–1193	-	
CK-NAC, 0–171 U/L	S	71 (92%)	105.4	22.5–2567	57.7–252.8	<0.0001
	D	14 (58%)	636.9 ****	85.6–2408	166.3–1061	
CK-MB, 0–24 U/L	S	72 (94%)	12.7	3.5–101.1	9.1–17.2	<0.0001
	D	15 (63%)	29.8 ****	10.8–201.5	19.1–64.3	
Troponin, 0–342 pg/mL	S	40 (52%)	3.05	0.10–20,019	1.65–7.35	0.0030
	D	7 (29%)	152.1 **	1.6–50,000	9.9–2317	
Procalcitonin (PCT), 0–0.046 ng/mL ^#^	S	7 (9%)	0.060	0.020–0.580	0.020–0.130	0.0208
	D	14 (58%)	0.210 *	0.050–3.690	0.108–0.725	
CRP 0–5 mg/mL	S	77 (100%)	49.5	0.67–292.7	17.5–107.5	0.3189
	D	24 (100%)	59.6	2.2–204.4	20.2–143.5	

^#^ <0.5 ng/mL procalcitonin, low probability of sepsis and/or septic shock; >2.0 ng/mL, high probability of sepsis and/or septic shock; *, differences are statistically significant, *p* < 0.05; **, differences are statistically significant, *p* < 0.01; ***, differences are statistically significant, *p* < 0.001; ****, differences are statistically significant, *p* < 0.0001.

**Table 3:** Esterase status of patients upon admission to the hospital.

Biochemical Values, Normal Ranges	Outcome	n	Median	Range (Min to Max)	Interquartile Range (1–3 Quartiles)	*p* Value
BChE activity with ATCh, µmol min^−1^ L^−1^	S	63 (82%)	1145	307–2172	836–1286	0.0083
	D	22 (92%)	832 **	249–1796	505–1168	
BChE activity with BTCh, µmol min^−1^ L^−1^	S	63 (82%)	2317	664–4775	1761–2564	0.0002
	D	22 (92%)	1471 ***	422–3266	944–2000	
PON1 activity, mmol min^−1^ L^−1^	S	63 (82%)	26.80	1.28–55.20	22.84–32.90	0.0924
	D	22 (92%)	24.28	4.40–36.60	17.78–31.12	
Esterase activity of albumin with NPA, µmol min^−1^ L^−1^	S	63 (82%)	13.60	6.00–523.50	6.00–59.70	>0.9999
	D	22 (92%)	13.47	6.00–246.80	6.00–54.65	
MDA, µmol/L	S	63 (82%)	2.00	0.10–7.87	1.30–3.20	0.0053
	D	22 (92%)	3.05 **	0.90–8.37	2.18–5.80	

Column n indicates the absolute number of patients with available data and the relative number (out of 77 survivors and 24 deceased); **, *p* < 0.01; ***, *p* < 0.001.

**Table 4:** New diagnostic indices. Data are presented as median and range from minimum to maximum. AGWF—vWF antigen; ActWF—vWF activity; BChEa—activity of BChE with ATCh as substrate; BChEb—BChE activity with BTCh as substrate; Ferr—ferritin; ALB—albumin. Concentration indicators are enclosed in square brackets, while enzymatic activity indicators are without brackets. *, differences are statistically significant, *p* < 0.05; **, differences are statistically significant, *p* < 0.01; ***, differences are statistically significant, *p* < 0.001; ****, differences are statistically significant, *p* < 0.0001.

Index	Survived	Deceased	*p* Value
[Urea] × [AGWF] × 1000/(BchEb × [ALB])	0.21 (0.04; 1.70) n = 37	1.54 (0.21; 23.63) **** n = 15	<0.0001
[Urea] × ActWF × 1000/(BchEb × [ALB])	11.35 (0.47; 81.57) n = 37	71.85 (2.30; 1435.00) *** n = 15	0.0001
[AGWF] × 10,000/(BchEb × [ALB])	0.39 (0.10; 1.37) n = 38	1.35 (0.26; 4.69) **** n = 15	<0.0001
ActWF × 1000/(BchEb × [ALB])	2.31 (0.09; 8.72) n = 38	7.93 (0.28; 33.06) ** n = 15	0.0036
[AGWF] × [Ferr] × 1000/(BchEb × [ALB])	16.78 (0.20; 127.80) n = 34	95.51 (6.26; 459.60) * n = 8	0.0286
ActWF × [Ferr] × 10/(BchEb × [ALB])	11.65 (0.12; 78.61) n = 34	74.39 (0.68; 279.00) n = 8	0.0600
Indices without data on vWF activity and concentration			
[Urea] × 10/[ALB]	1.41 (0.49; 5.98) n = 38	4.26 (1.62; 20.20) **** n = 15	<0.0001
[MDA] × 100/[ALB]	5.54 (0.62; 25.56) n = 38	12.20 (6.98; 27.14) ** n = 15	0.0010
BChEa/[ALB]	30.18 (13.92; 46.85) n = 38	24.93 (12.38; 39.47) n = 15	0.0916
BChEb/[ALB]	60.21 (21.45; 89.35) n = 38	47.09 (25.51; 65.38) ** n = 15	0.0012
[Creatinine] × [MDA]/[ALB]	5.06 (0.47; 26.25) n = 38	8.45 (2.14; 53.08) * n = 15	0.0255
[Urea] × [MDA]/[ALB]	0.28 (0.01; 2.08) n = 38	1.38 (0.54; 13.71) **** n = 15	<0.0001
BChEa/[MDA]	514 (101; 9098) n = 63	195 (59; 898) *** n = 21	0.0001
BChEb/[MDA]	1021 (251; 17,648) n = 63	382 (99; 1705) **** n = 21	<0.0001
[Urea] × [MDA] × 1000/(BChEb × [ALB])	0.14 (0.01; 0.96) n = 37	1.24 (0.32; 10.36) **** n = 15	<0.0001
[Urea] × 1000/(BChEb × [ALB])	0.06 (0.02; 0.27) n = 37	0.24 (0.09; 1.59) **** n = 15	<0.0001

**Table 5:** Prognostic values of some single and novel derived diagnostic indices.

Biochemical Values or Index, n	AUC, (95% CI)	*p* Value	Critical Value	Likelihood Ratio	OR, (95% CI)	*p* Value
Troponin, 47	0.84 (0.64–1.00)	0.0045	>51.45	14.3	47.5 (5.4–416)	0.0005
Procalcitonin, 21	0.81 (0.60–1.00)	0.02	>0.145	5.0	15.0 (1.3–168)	0.028
CRP, 101	0.57 (0.43–0.70)	0.32	>167.0	3.2	3.7 (0.8–15.9)	0.085
[Urea] × [AGWF] × 1000/ (BChEb × [ALB]), 52	0.91 (0.83–1.00)	<0.0001	>0.96	24.7	72.0 (7.5–689)	0.0002
[Urea] × ActWF × 1000/ (BChEb × [ALB]), 52	0.83 (0.68–0.97)	0.0002	>63.23	22.2	72.0 (7.5–689)	0.0002
[AGWF] × 10,000/(BChEb × [ALB]), 53	0.86 (0.73–0.98)	<0.0001	>1.20	22.8	55.50 (5.9–521)	0.0004
ActWF × 1000/ (BChEb × [ALB]), 53	0.75 (0.56–0.93)	0.0059	>7.77	11.4	27.00 (4.7–157)	0.0002
[Urea] × 10/[ALB], 53	0.94 (0.87–1.00)	<0.0001	>3.85	20.3	42.3 (4.6–393)	0.001
[Urea] × [MDA]/[ALB], 52	0.92 (0.84–0.99)	<0.0001	>2.00	14.8	24.0 (2.6–225)	0.0054
[Urea] × [MDA] × 1000/ (BChEb × [ALB]), 52	0.94 (0.89–1.00)	<0.0001	>0.945	19.7	41.1 (4.4–383)	0.0011
[Urea] × 1000/(BChEb × [ALB]), 52	0.95 (0.89–1.00)	<0.0001	>0.265	17.3	31.5 (3.4–293)	0.0024

## Data Availability

The data presented in this study are available from the corresponding authors upon reasonable request. Data is not publicly available due to privacy.

## Abbreviations

ActWF	vWF activity;
ADAMTS13	disintegrin and metalloproteinase with a thrombospondin type 1 motif, member 13;
AGWF	vWF antigen;
ALB	albumin;
ALP	alkaline phosphatase;
ALT	alanine aminotransferase;
ARDS	acute respiratory distress syndrome;
AST	aspartate aminotransferase;
ATCh	acetylthiocholine;
AUC	area under the curve;
BChEa	BChE activity with ATCh;
BChEb	BChE activity with BTCh;
BTCh	butyrylthiocholine;
BChE	butyrylcholinesterase;
CIC	coronavirus-induced coagulopathy;
CT	computed tomography;
CK-NAC	total creatine phosphokinase;
CK-MB	muscle brain isoform of creatine phosphokinase;
CRP	C-reactive protein;
DIC	disseminated intravascular coagulation;
DLCO	carbon monoxide diffusion capacity of the lungs;
EC	endothelial cells;
Ferr	ferritin;
FEV1	forced expiratory volume in the first second;
FVC	forced volume capacity;
GGT	gamma-glutamyltransferase;
HUS	hemolytic-uremic syndrome;
IL-6	interleukin 6;
ICU	intensive care unit;
LDH	lactate dehydrogenase;
LR	likelihood ratio;
MAP	microangiopathy;
MDA	malondialdehyde;
MODS	multiple organ dysfunction syndrome;
NPA	nitrophenylacetate;
OR	odds ratio;
PE	pulmonary embolism;
PON1	paraoxonase 1;
ROS	reactive oxygen species;
SO2	oxygen saturation;
TTP	thrombotic thrombocytopenic purpura;
Urea	urea;
vWF	von Willebrand factor;
WPBs	Weibel–Palade bodies.
